# A Method of Operating about the Face, by Which Little Blood Enters the Mouth

**Published:** 1893-07

**Authors:** W. W. Keen


### A METHOD OF OPERATING ABOUT THE FACE BY WHICH BUT LITTLE BLOOD ENTERS THE MOUTH.

BY W. W. KEEN, M. D.

I wish to call attention to a method of operating about the face which is not generally followed, and which may be new to some. It is particularly applicable to epithelioma about the lip, of which I have had two severe cases in a comparatively short time. In one there was extensive epithelioma requiring removal of a portion of the upper lip, the lower lip, and the cheek. The other case was one of epithelioma of the cheek, and is also of interest from the fact that the patient had originally been operated on forty years ago by Dr. George R. Morehouse. A microscopic examination was made at that time, and the tumor was said not to be epithelioma. When I saw the man the disease extended from the angle of the mouth back to the molar teeth, and from one jaw to the other. It was on the inside of the mouth exclusively, except at the angle where the entire thickness was involved. The external layer of the cheek seemed to be entirely free. In operating I placed the patient on a flat table with the operating side turned a little down and cut through the skin down to the mucous membrane, but not through the latter. I then secured all the vessels *before* opening into the mouth. In this way I prevented blood from entering the mouth and also lessened the total loss of blood. In this case Stenson's duct was involved in the operation. I found the duct and stitched it to the mucous membrane of the upper jaw, and there has not been the slightest trouble from fistula. The incision was a very wide V, the linear incision corresponding to the apex of the V being on the cheek, and the widest part in the base of the V inside the mouth. I am sure that those of you who try this method will find it satisfactory.

